# Kernel Sparse Representation with Hybrid Regularization for On-Road Traffic Sensor Data Imputation

**DOI:** 10.3390/s18092884

**Published:** 2018-08-31

**Authors:** Xiaobo Chen, Cheng Chen, Yingfeng Cai, Hai Wang, Qiaolin Ye

**Affiliations:** 1 Automotive Engineering Research Institute, Jiangsu University, Zhenjiang 212013, China; yfcai@ujs.edu.cn; 2 School of Automotive and Traffic Engineering, Jiangsu University, Zhenjiang 212013, China; 13851301126@126.com (C.C.); 1000004061@ujs.edu.cn (H.W.); 3 College of Information Science and Technology, Nanjing Forestry University, Nanjing 210037, China; yqlcom@njfu.edu.cn

**Keywords:** sparse representation, elastic net, kernel method, missing data, imputation

## Abstract

The problem of missing values (MVs) in traffic sensor data analysis is universal in current intelligent transportation systems because of various reasons, such as sensor malfunction, transmission failure, etc. Accurate imputation of MVs is the foundation of subsequent data analysis tasks since most analysis algorithms need complete data as input. In this work, a novel MVs imputation approach termed as kernel sparse representation with elastic net regularization (KSR-EN) is developed for reconstructing MVs to facilitate analysis with traffic sensor data. The idea is to represent each sample as a linear combination of other samples due to inherent spatiotemporal correlation, as well as periodicity of daily traffic flow. To discover few yet correlated samples and make full use of the valuable information, a combination of *l*_1_-norm and *l*_2_-norm is employed to penalize the combination coefficients. Moreover, the linear representation among samples is extended to nonlinear representation by mapping input data space into high-dimensional feature space, which further enhances the recovery performance of our proposed approach. An efficient iterative algorithm is developed for solving KSR-EN model. The proposed method is verified on both an artificially simulated dataset and a public road network traffic sensor data. The results demonstrate the effectiveness of the proposed approach in terms of MVs imputation.

## 1. Introduction

With the rapid development of finance and society, traffic congestion has become an urgent worldwide problem causing the waste of travel time, environmental pollution, etc. To alleviate traffic congestion and improve road capacity, intelligent transportation system (ITS) [1] was developed by integrating various techniques, such as computer, communication, artificial intelligence, and so on. Data acquisition through different sensors is the foundation of ITS, where higher-level functions including traffic forecasting, route planning, etc., usually depend on the quality and quantity of sensor data. Unfortunately, traffic data acquired by sensors are usually incomplete, indicating the loss of many entries, due to various unpredictable factors, including sensor malfunction, transmission network anomaly, storage equipment damage, etc. For example, for a dense road network in Melbourne city, about 8% of its sensors can reach up to 56% missing data. Likewise, about 10% of daily traffic volume in Beijing is missing [2], and according to Turner et al. [3], more than 5% of data within the PeMS database are lost. The loss of traffic sensor data is unavoidable under existing technical level and objective conditions, therefore posing great challenge for effective deployment of ITS. For instance, most of the traffic state forecasting algorithms [4], either statistical approaches such as support vector regression (SVR) [5,6,7] and neural networks (NNs) [8,9,10], or model based approaches [11,12,13,14] such as extended Kalman filter (EKF) [13] and Hamilton-Jacobi equations [12], cannot leverage incomplete data for model training without proper preprocessing.

During the last few decades, data imputation has drawn much attention of researchers because it provides a rational way to make use of incomplete data. Here, imputation or recovery refers to the procedure providing plausible estimations for those missing values (MVs) given other observed values. After imputation, original incomplete data can be converted into complete data and then used in subsequent data analysis tasks. It is feasible to obtain accurate estimation of MVs because data generated in real-world usually possess inherent structure such that close relationship between MVs and observed values can be reliably established based on a set of incomplete samples. In traffic scenarios, due to the connectivity of road segments and the periodicity of people’s travel behavior, traffic flow data acquired by different sensors installed in the same road network often have strong spatiotemporal correlation. For instance, traffic flow in different days of a week shows a certain degree of similarity. The upstream traffic and the neighboring downstream traffic will drastically influence each other. As a result, there exist underlying structures hidden from the raw high-dimensional sensor data. Until now, many MVs imputation algorithms have been developed in literatures, including mean imputation (MI), K-nearest neighbor (KNN) [15], support vector machine (SVM) [16], singular value decomposition (SVD) [17], probability principal component analysis (PPCA) [2], low-rank matrix completion (LRMC) [18,19], etc.

Recently, a sample self-representation based method was put forward [20] to recover MVs from incomplete data. Different from the above algorithms, it assumes that original data are derived from multiple low-dimensional linear subspaces [21,22]. In such a case, each sample can be well reconstructed by linearly combining a few of the other samples that belong to the same subspace. Then, MVs can be recovered such that the discrepancy between the observed values and their reconstructed values, namely the reconstruction error, should be as small as possible. In addition, *l*_1_-norm and *l*_2_-norm are utilized as regularization on combination coefficients (weights) to avoid possible overfitting. It also reported that *l*_1_-norm is more preferable than *l*_2_-norm in terms of recovery performance, which is consistent with the advantages of sparse representation (SR) models [21,23], where each sample can be sparsely instead of densely reconstructed by other samples within the same class. Overall, this method has demonstrated improved imputation performance in comparison with other competing algorithms [24,25], thus confirming to some extent the rationality of SR in MVs imputation. However, SR based on either *l*_1_-norm or *l*_2_-norm may fail to take full advantage of all the information contained in samples. It has been revealed that *l*_1_-norm, despite sparsity-inducing characteristic, tends to select only one variable from a group of highly correlated variables [26]. It may not work well for the case where samples are highly correlated to each other. From another point of view, performing SR in the input data space potentially assumes linear relations among data and thus may fail to analyze data with complex nonlinear structure [27,28]. It is seldom that data in the actual environment, such as traffic sensor data, completely conform to such linear relations. As a consequence, the existing SR based method may lead to suboptimal imputation performance when this prerequisite is violated.

Motivated by the above observations, we propose in this paper a novel MVs imputation approach termed as kernel sparse representation with elastic net regularization (KSR-EN), which is applied to road network traffic sensor data. Our work is interesting from the following aspects:

- We perform MVs imputation in kernel-induced high-dimensional feature space instead of the original input space [29,30]. By doing so, nonlinear relationship among data samples can be discovered and leveraged for recovery performance improvement. To the best of our knowledge, this is the first work to which KSR was applied for MVs imputation.
- We propose to apply the combination of *l*_1_-norm and *l*_2_-norm, namely elastic net in statistics literature [26], as regularization on the representation coefficients, with the hope that enough information can be extracted from those highly correlated samples for recovering MVs.
- An iterative algorithm is developed for solving the resulting KSR-EN model by integrating monotone fast iterative shrinkage thresholding algorithm (FISTA) [31,32,33], as well as projected gradient descent (PGD) approach [24].
- The proposed model is evaluated on both synthetic data and real-work traffic sensor data. The results demonstrate that KSR-EN outperforms other competing algorithms in terms of MVs imputation.

The remainder of this paper is organized as follows. In Section 2, we briefly review some popular approaches for MVs imputation. In Section 3, KSR-EN model and corresponding solution algorithm are proposed. Section 4 reports some experiments on simulated as well as real-world traffic sensor data. Finally, we give some conclusions and future works in Section 5.

## 2. Related Work

In order to solve the problem of MVs imputation, many algorithms have been put forward by researchers from different perspectives. The most widely applied approaches can be roughly divided into three categories which are discussed in sequel.

### 2.1. Probabilistic Model Based Methods

For this type of method, a statistical model responsible for producing complete data needs to be specified. One of the most common choices is the multivariate Gaussian model and its extension [34]. Such a model is used to describe the inherent relationship between variables, forming the basis for MVs recovery. The model parameters, along with those MVs, can be estimated simultaneously via alternating optimization. In probabilistic principal component analysis (PPCA) [2,35], the data is assumed to be drawn from a single low-dimensional linear subspace. Then, the likelihood of observed values is derived and maximized through the well-established expectation maximization (EM) algorithm [36]. Furthermore, Bayesian PCA [37] combines the advantages of Bayesian learning [38] and PPCA such that the dimensionality of latent space in PPCA can be automatically inferred based on data. These methods are suitable for data with dominant structure. However, PPCA may perform poorly when missing ratio in data is high [39]. Moreover, how to specify a proper statistical model for real-world data is not a trivial task. In practice, it may be infeasible to postulate a uniformed model for different types of data.

### 2.2. Regression Model Based Methods

The regression-based methods attempt to establish regression equation to characterize the relationship between a number of observed variables and other variables with MVs based on a set of samples. Various concrete regression techniques can be adopted for this purpose, such as linear regression, support vector machine (SVM) [40,41], and neural networks (NNs) [42]. Then, MVs are replaced with the conditional expectation of the regression results. Local least squares (LLS) regression is a typical regression based imputation method which has been successfully applied in different situations [43]. By using K-nearest neighboring search, LLS first chooses a small number of variables which are most similar to the target variable. Then, least squares criterion is used to establish the regression equation based on the observed data. By doing so, LLS succeeds in exploiting underlying local similarity structure among data and thus is more applicable when data distribute in a nonlinear way. However, LLS may fail in the case of high missing ratio due to unreliable estimation of nearest neighbors or improper regression model.

### 2.3. Matrix Completion Based Methods

As stated in Section 1, traffic sensor data in the same road network usually exhibit strong spatiotemporal correlation because of road structure, as well as people’s travel behavior. As a consequence, the data matrix usually has low-rank property, implying the number of independent rows (and columns) is much smaller than the size of matrix. Under such a circumstance, low-rank matrix completion (LRMC) [18,19] can be used to recover MVs through rank (or its surrogate nuclear norm) minimization on the whole matrix. Many efficient optimization algorithms have been developed to solve the problem, e.g., SVT [18,44], FPCA [45], ADMM [46], etc. However, LRMC depends on global linear correlation, which is restricted for real data, such as traffic sensor data [19]. Recently, sparse representation (SR) [23] based subspace clustering [21] has drawn much attention because it supposes that the samples are drawn from a union of multiple subspaces, instead of a single one. Due to such advantage, it can reveal complex structure of data, thus leading to better imputation performance [20]. Nevertheless, many datasets in practice are not necessarily well characterized by multiple linear models, and in such cases, existing algorithms may produce suboptimal imputation results [47].

## 3. Kernel Sparse Representation with Elastic Net Regularization

### 3.1. Linear and Kernel SR-EN

Given data matrix $X=\left[x_{1},x_{2},\cdots ,x_{N}\right]\in R^{p\times N}$, where sample $x_{i}={\left[x_{i}\left(1\right),x_{i}\left(2\right),\cdots ,x_{i}\left(p\right)\right]}^{T}\in R^{p}$, p and N denote the number of features and samples, respectively. In the case we study, not all of the entries in sample $X_{i}$ are known. Therefore, let Ω indicate the index set of observed entries in X, that is, for all (i,j)∈Ω, $x_{i}\left(j\right)$ is observable. The task here is to obtain an accurate estimation for those $x_{i}\left(j\right)$, (i,j)∉Ω. Sharing similar flavor with SR [23] and sparse subspace clustering [21,48], we hope that each sample can be well approximated as a linear combination of other samples, i.e., $x_{i}\approx \overset{N}{\underset{j=1}{\sum }}x_{j}w_{i}\left(j\right)$ where $w_{i}\left(j\right)$ is an element in coefficient matrix $W\in R^{N\times N}$, indicating the contribution of $x_{j}$ in reconstructing $x_{i}$. Additionally, the constraint $w_{i}\left(i\right)=0$, i.e., diag(W)=0 in matrix form, is added to avoid trivial solution. Besides this reconstruction criterion, we also impose a penalty to W so as to alleviate possible overfitting. In this work, elastic net regularization [26], as a hybrid of *l*_1_-norm and *l*_2_-norm, is adopted since it not only achieves sparse variable selection but also would benefit from highly correlated variables. By integrating the above ingredients together, we propose the following MVs imputation model termed as SR with elastic net regularization (SR-EN):

(1) $$\underset{X,W}{\min}\frac{1}{2}{\left\|X-XW\right\|}^{2}+C\alpha {\left\|W\right\|}_{1}+\frac{C\left(1-\alpha \right)}{2}{\left\|W\right\|}^{2}\;s.t.\;\mathrm{diag}(W)\;=0,\;x_{i}\left(j\right)=m_{i}\left(j\right),\;\left(i,j\right)\in \Omega$$

where ${\left\|W\right\|}_{1}=\overset{N}{\underset{i=1}{\sum }}\overset{N}{\underset{j=1}{\sum }}\left|w_{i}\left(i\right)\right|$, ${\left\|W\right\|}^{2}=\overset{N}{\underset{i=1}{\sum }}\overset{N}{\underset{j=1}{\sum }}{\left(w_{i}\left(i\right)\right)}^{2}$, $m_{i}\left(j\right)$ is the observed value for $x_{i}\left(j\right)$, C>0, and 1≥α≥0 are two parameters used to balance the role of regularization and *l*_1_-norm, respectively. When α=1 or α=0, elastic net regularization will degenerate to pure *l*_1_-norm or *l*_2_-norm regularization.

The above SR-EN model is able to recover missing values when samples distribute in a union of multiple linear subspaces. However, it may produce suboptimal imputation when applied to data with nonlinear structure. Therefore, we further extend SR-EN to deal with samples distributed nonlinearly in raw input space. To achieve this goal, motivated by kernel method [5], we first map the original input space $R^{p}$ to a reproducing kernel Hilbert space (RKHS) H with higher or even infinite dimensionality, by employing nonlinear mapping function $\phi :R^{p}\to H$. Let ϕ(x)∈H be the image of sample x in feature space H, and $\phi \left(X\right)=\left[\phi \left(x_{1}\right),\phi \left(x_{2}\right),\cdots ,\phi \left(x_{N}\right)\right]$ denotes the entire sample matrix after mapping. Similar to classic kernel-based learning, we make an assumption that nonlinear distribution of a given dataset in original input space $R^{p}$ can be well converted into linear distribution in feature space H with much higher dimensionality, and thus facilitating the application of SR-EN. Based on the above discussion, the kernel SR-EN (KSR-EN) we propose can be expressed as:

(2) $$\underset{X,W}{\min}\frac{1}{2}{\left\|\phi \left(X\right)-\phi \left(X\right)W\right\|}^{2}+C\alpha {\left\|W\right\|}_{1}+\frac{C\left(1-\alpha \right)}{2}{\left\|W\right\|}^{2}\;s.t.\;\mathrm{diag}\left(W\right)=0,\;x_{i}\left(j\right)=m_{i}\left(j\right),\;\left(i,j\right)\in \Omega$$

**Figure 1:**
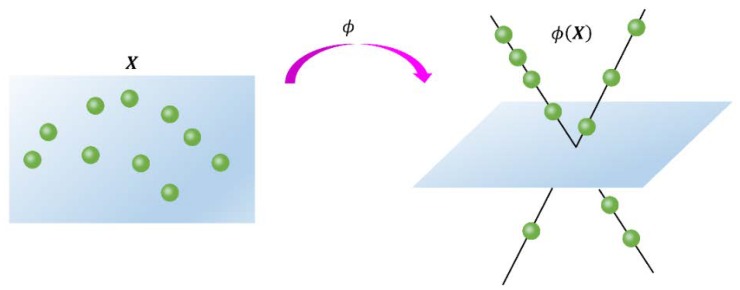
Nonlinear mapping in the proposed method.

Figure 1 presents the effect of nonlinear mapping. Note that the above KSR-EN model reduces to its linear version (1) when the mapping function ϕ is linear, namely, ϕ(x)=x. Therefore, in what follows, we will concentrate on KSR-EN (2) and develop an effective algorithm for solving it. We let $\lambda_{1}=C\alpha$ and $\lambda_{2}=C\left(1-\alpha \right)$ to simplify notations when deriving optimization algorithm.

### 3.2. Optimization Algorithm

As we can see from (2), the coupling between decision variables X and W makes it difficult to find optimal solutions for X and W simultaneously. Therefore, in this work, we choose alternating optimization scheme to find the optimal solution iteratively. Specifically, we attempt to solve (2) by alternatively optimizing over W and X while holding the other variable fixed.

Optimize W while fixing X. In such a case, (2) leads to the following optimization problem:

(3) $$\underset{W}{\min\;}\frac{1}{2}{\left\|\phi \left(X\right)-\phi \left(X\right)W\right\|}^{2}+\lambda_{1}{\left\|W\right\|}_{1}+\frac{\lambda_{2}}{2}{\left\|W\right\|}^{2}$$

In terms of our problem (3), the objective can be decomposed into the sum of two parts f(W)+g(W), where $f\left(W\right)=\frac{1}{2}{\left\|\phi \left(X\right)-\phi \left(X\right)W\right\|}^{2}$ and $g\left(W\right)=\lambda_{1}{\left\|W\right\|}_{1}+\frac{\lambda_{2}}{2}{\left\|W\right\|}^{2}$. Let us focus on the first part, f(W). It is obvious that f(W) is convex and differentiable and can be reformulated as $f\left(W\right)=\frac{1}{2}\mathrm{tr}\left(\phi {\left(X\right)}^{T}\phi \left(X\right)-\phi {\left(X\right)}^{T}\phi \left(X\right)W-W^{T}\phi {\left(X\right)}^{T}\phi \left(X\right)+W^{T}\phi {\left(X\right)}^{T}\phi \left(X\right)W\right)$, where tr(·) denotes the trace operator of a matrix. Furthermore, based on the rule of matrix derivative [49], the gradient of f(W) w.r.t. W is given by $\nabla f\left(W\right)=\phi {\left(X\right)}^{T}\phi \left(X\right)W-\phi {\left(X\right)}^{T}\phi \left(X\right)$. We notice immediately that both f(W) and its gradient, ∇f(W), depend exclusively on the inner products between the images of all pairs of samples in feature space, without having to give explicit representation for ϕ. In other words, we can introduce kernel Gram matrix $K=\phi {\left(X\right)}^{T}\phi \left(X\right)$, with element $K_{ij}$ computed as $K_{ij}=\phi {\left(x_{i}\right)}^{T}\phi \left(x_{j}\right)$. Then, we have:

(4) $$f\left(W\right)=\frac{1}{2}\mathrm{tr}\left(K-KW-W^{T}K+W^{T}KW\right)\;\mathrm{and}\;\nabla f\left(W\right)=KW-K$$

According to Mercer’s theorem [50], any matrix K could be a valid kernel as long as it is positive semi-definite (PSD). Some commonly used kernels include polynomial kernel, radial basis function (RBF) kernel, sigmoid kernel, etc. In this work, RBF kernel, defined as $K_{ij}=\phi {\left(x_{i}\right)}^{T}\phi \left(x_{j}\right)=e^{-\gamma {\left\|x_{i}-x_{j}\right\|}^{2}}$, is used because of its simplicity, along with good empirical performance in various kernel-based learning algorithms. In RBF kernel, γ is a free parameter controlling the smoothness degree of the kernel.

On the other hand, the second part, g(W), i.e., the regularization term, is convex yet nondifferentiable, thus restricting the application of traditional gradient descent algorithm. Nevertheless, considering the facts that ∇f(W) is Lipschitz-continues and g(W) has a closed-form proximity operator, we develop a first-order algorithm to find the optimal solution of (3) under the proximal gradient descent framework. This framework, also known as fast iterative shrinkage thresholding approach (FISTA), has been widely used to solve various sparsity-related problems, such as [33].

Specifically, given the Lipschitz constant L of ∇f(W) and the current solution $W_{k}$ of (3) at the k-th iteration, it is possible to construct an approximating function $q\left(W,W_{k}\right)$ majorizing the original f(W) at $W_{k}$:

(5) $$q\left(W,W_{k}\right)=f\left(W_{k}\right)+\langle W-W_{k},\nabla f(W_{k})\rangle +\frac{L}{2}{\left\|W-W_{k}\right\|}^{2}+g\left(W\right)$$

where the definitions of $f\left(W_{k}\right)$ and $\nabla f\left(W_{k}\right)$ are given in (4). From the definition of $q\left(W,W_{k}\right)$, we have:

(6) $$q\left(W,W_{k}\right)\geq f\left(W\right),\;\forall W$$

where the equality holds if and only if $W=W_{k}$. This fact motivates the following update:

(7) $$W_{k+1\;}=\arg\underset{W}{\min}q\left(W,W_{k}\right)$$

We can prove that $W_{k+1}$ will lead to an improved objective value for (3) because:

(8) $$f\left(W_{k}\;\right)=q\left(W_{k},W_{k}\right)\geq q\left(W_{k+1},W_{k}\right)\geq f\left(W_{k+1}\right)$$

In order to solve (7), we rewrite the objective (5) as:

(9) $$q\left(W,W_{k}\;\right)\propto \frac{L}{2}{\left\|W-\left(W_{k}-\frac{\nabla f\left(W_{k}\right)}{L}\right)\right\|}^{2}+g\left(W\right)$$

Let $V_{k}=W_{k}-\frac{\nabla f\left(W_{k}\right)}{L}$ and incorporating the definition of g(W), $W_{k+1}$ can be obtained by:

(10) $$W_{k+1\;}=\arg\underset{W}{\min}\frac{L}{2}{\left\|W-V_{k}\right\|}^{2}+\lambda_{1}{\left\|W\right\|}_{1}+\frac{\lambda_{2}}{2}{\left\|W\right\|}^{2}$$

Notice that all of the entries in W are independent of each other, and thus can be optimized separately and parallelly. For the sake of simplicity, consider the proximity operator for elastic net regularization with univariate as follows:

(11) $$\mathrm{Prox}(b)\;=\arg\underset{a}{\min}\frac{1}{2}{\left(a-b\right)}^{2}+\lambda_{1}\left|a\right|+\frac{\lambda_{2}}{2}a^{2}$$

where a, b∈R. Problem (11) has a closed-form optimal solution as:

(12) $$\mathrm{Prox}(b)\;=\mathrm{sign}\left(b\right){\left(\frac{\left|b\right|-\lambda_{1}}{1+\lambda_{2}}\right)}_{+}$$

where ${\left(a\right)}_{+}=\underset{}{\max}\left\{0,a\right\}$. Incorporating the above result into (10), one can obtain the solution $W_{k+1}$ as:

(13) $$W_{k+1\;}=\mathrm{sign}\left(V_{k}\right){\left(\frac{L\left|V_{k}\right|-\lambda_{1}}{L+\lambda_{2}}\right)}_{+}$$

The whole algorithm for solving (3) is summarized in Algorithm 1.

**Algorithm 1.** Monotone fast iterative shrinkage thresholding algorithm for solving (3)**Input**: Estimated data matrix X, trade-off parameters $\lambda_{1}$, $\lambda_{2}$, Gaussian kernel parameter γ
Initialize $W_{0}$**Output**: Coefficient matrix W
**Procedure**
Calculate N×N kernel matrix K with $K_{ij}=e^{-\gamma {\left\|x_{i}-x_{j}\right\|}^{2}}$, $L=1.1{\left\|K\right\|}_{2}$, $Y_{1}=W_{0}$, k=1, $t_{1}=1$**while** not converge **do**
$Z_{k}=Y_{k}-\frac{KY_{k}-K}{L}$

$Z_{k}=\mathrm{sign}\left(Z_{k}\right){\left(\frac{L\left|Z_{k}\right|-\lambda_{1}}{L+\lambda_{2}}\right)}_{+}$

$\mathrm{diag}\left(Z_{k}\right)=0$

$t_{k+1}=\frac{1+\sqrt{1+4t_{k}^{2}}}{2}$

$W_{k}=\underset{W}{\min}f\left(W\right)+g\left(W\right)\;s.t.\;W=Z_{k},W_{k-1}$

$Y_{k+1}=W_{k}+\left(\frac{t_{k}}{t_{k+1}}\right)\left(Z_{k}-W_{k}\right)+\left(\frac{t_{k}-1}{t_{k+1}}\right)\left(W_{k}-W_{k-1}\right)$

k=k+1

**end while**


Optimize X while fixing W. In such a case, (2) leads to the following optimization problem with equality constraint:

(14) $$\underset{X}{\min}\mathrm{tr}\left(K-KW-W^{T}K+W^{T}KW\right)\;s.t.\;x_{i}\left(j\right)=m_{i}\left(j\right),\;\left(i,j\right)\in \Omega$$

where we have used the results in (4). To solve (14), we employ the projected gradient descent method with Armijo step size rule. The essential ingredient is the calculation of derivative w.r.t. decision variables.

Denoting the objective in (14), as $h\left(X\right)=\mathrm{tr}\left(K-KW-W^{T}K+W^{T}KW\right)$, its partial derivative w.r.t. the kernel matrix K is given by:

(15) $$\frac{\partial h\left(X\right)}{\partial K}=\left(I-W\right){\left(I-W\right)}^{T}$$

Furthermore, for RBF kernel $K_{ij}=e^{-\gamma {\left\|x_{i}-x_{k}\right\|}^{2}}$, we can easily compute:

(16) $$\frac{\partial K_{ik\;}}{\partial x_{i}}=-2\gamma \left(x_{i}-x_{k}\right)e^{-\gamma {\left\|x_{i}-x_{k}\right\|}^{2}}$$

To calculate the derivative of h(X) w.r.t. the entries in X, we apply the chain rule to get:

(17) $$\frac{\partial h\left(X\right)}{\partial x_{i}\left(j\right)}=\underset{k}{\sum }\frac{\partial h\left(X\right)}{\partial K_{ik}}\frac{\partial K_{ik}}{\partial x_{i}\left(j\right)}$$

where $\frac{\partial h\left(X\right)}{\partial K_{ik}}$ and $\frac{\partial K_{ik}}{\partial x_{i}\left(j\right)}$ can be obtained from (15) and (16), respectively. For clarity, the algorithm for solving (14) is presented in Algorithm 2.

**Algorithm 2.** Gradient descent algorithm for solving (14)**Input**: Coefficient matrix W**Output**: Estimated data matrix X
**Procedure**
Initialize data matrix X
**while** not converge **do**Compute the derivative of h(X) w.r.t. X, that is $\frac{\partial h\left(X\right)}{\partial X}$ using (17)Let $\frac{\partial h\left(X\right)}{\partial x_{i}\left(j\right)}=0$, for any (i,j)∈ΩFind step size l with Armijo rule, that is, choose $l=\underset{}{\max}\left\{1,\frac{1}{2},\frac{1}{4},\cdots \right\}$ such that 
$h\left(X\right)-h\left(X-l\frac{\partial h\left(X\right)}{\partial X}\right)\geq \frac{l}{4}{\left\|\frac{\partial h\left(X\right)}{\partial X}\right\|}^{2}$
Update $X=X-l\frac{\partial h\left(X\right)}{\partial X}$
**end while**


## 4. Experiments

### 4.1. Configuration

Besides the proposed KSR-EN (and its linear version SR-EN), we include some typical imputation algorithms including LLS, PPCA, and LRMC in order to comprehensively evaluate their performance. As stated in Section 2, LLS is a regression based method, PPCA relies on statistical assumption of data, and LRMC imposes low-rank property of sample matrix. Some recent studies [2,51] have manifested that PPCA and LRMC are two effective approaches for traffic sensor data. All of these methods are implemented in MATLAB 2015a on a PC with Core i7 2.4 GHz CPU and 12GB RAM. Following [20,24], the parameters in each method, such as the number of K-nearest neighbors in LLS, the dimensionality of latent space in PPCA, are tuned to give optimal performance.

To measure the accuracy of each method, we randomly produce MVs and then employ different methods to obtain corresponding estimations. Finally, the estimated values are compared with the real values and the difference between them are calculated. In this work, two widely applied metrics, i.e., root mean square error (RMSE) and relative error (RELERR), are calculated as follows:

(18) $$RMSE=\sqrt{\frac{1}{T}\underset{\left(i,j\right)\notin \Omega }{\sum }{\left(x_{i}\left(j\right)-{\hat{x}}_{i}\left(j\right)\right)}^{2}}$$

(19) $$RELERR=\sqrt{\frac{\sum_{\left(i,j\right)\notin \Omega }{\left(x_{i}\left(j\right)-{\hat{x}}_{i}\left(j\right)\right)}^{2}}{\sum_{\left(i,j\right)\notin \Omega }{\left(x_{i}\left(j\right)\right)}^{2}}}$$

where T denotes the total number of missing entries, and ${\hat{x}}_{i}\left(j\right)$ and $x_{i}\left(j\right)$ denote the imputed value and the real value, respectively. Obviously, the smaller the RMSE and RELERR, the better the imputation performance. In addition, we repeat each test 10 times and report the mean imputation error and the associated standard deviation, so as to reduce the potential bias caused by randomness.

### 4.2. Synthetic Data

**Figure 2:**
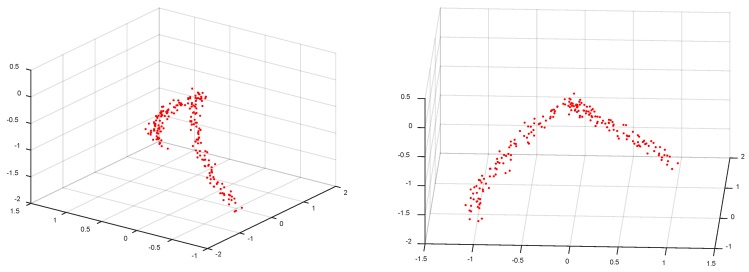
Illustration of simulated data from two different viewpoints.

**Figure 3:**
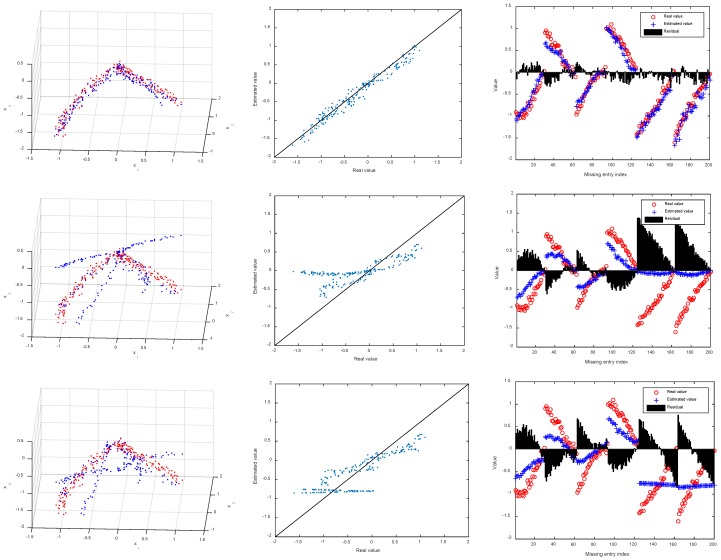

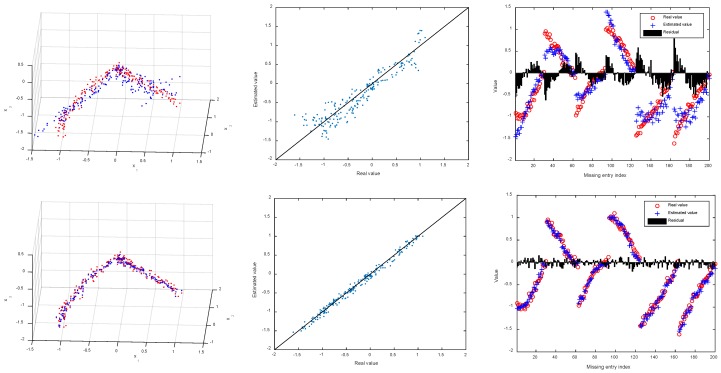
Imputation results obtained by local least squares (LLS), low-rank matrix completion (LRMC), probability principal component analysis (PPCA), sparse representation with elastic net regularization (SR-EN), and kernel sparse representation with elastic net regularization (KSR-EN) (from **top** to **bottom**).

We first evaluate the proposed method on nonlinear synthetic dataset to intuitively illustrate its behavior. The simulated samples are shown as the red points in Figure 2. The specific equations for generating these samples are described as follows:

$$\mathrm{Arc}\;1:\;x_{1}=sin\left(t\right),\;x_{2}=cos\left(t\right)-1,\;x_{3}=t\;\mathrm{Arc}\;2:\;x_{1}=1-cos(t)\;,\;x_{2}=-sin\left(t\right),\;x_{3}=t$$

where latent variable t is sampled from a one-dimensional uniform distribution in the interval $\left[-\frac{\pi }{2},0\right]$. As we can see, the entire set of samples is drawn from two arcs which intersect at the origin. The intrinsic dimension for each arc is 1. Gaussian noise with zero mean and 0.05 standard deviation is added to each sample. We synthesize 100 samples from each arc and the whole sample set, organized in matrix $X\in R^{3\times 200}$ in this case, forms a nonlinear structure in three-dimensional space. To get incomplete matrix, a randomly selected entry for each sample is removed, thus resulting in an incomplete matrix with missing ratio of 33.33%. Then, different imputation approach is applied to restore the missing entries of the data matrix. Figure 3 show the results in one experiment. Notice that in Figure 3, the first column depicts the real samples (red points) and the imputed samples (blue points), the second column shows the scatter plot of real and estimated values, and the last column further shows the residual. The averaged errors obtained by different methods across 10 tests are summarized in Table 1, where the best results are highlighted in bold.

**Figure 4:**
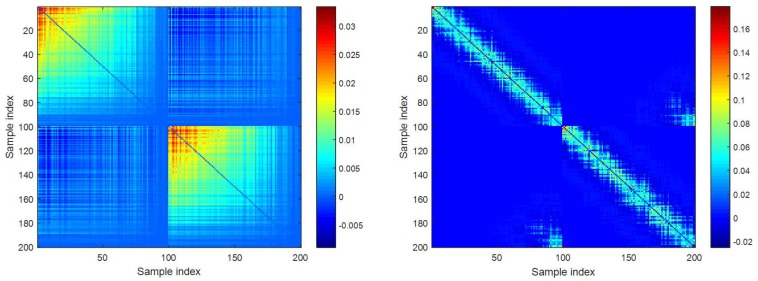
Illustration of coefficient matrix obtained by SR-EN (**left**) and KSR-EN (**right**).

As can be seen from these results, LRMC performs worst since it heavily depends on the global low-rank structure of data, which is violated in our case. Instead of minimizing the rank of data for recovery, PPCA makes use of maximum likelihood estimation and EM algorithm to jointly optimize model parameters and MVs. As a result, PPCA works better than LRMC, although it also imposes Gaussian distribution for data. SR-EN further improves the results of PPCA, although it is also based on linear structure of data. Different from PPCA, SR-EN is applicable to data lying on or close to a union of linear subspaces [48]. LLS achieves significantly smaller errors than LRMC, PPCA, and SR-EN. It may be because LLS is an imputation algorithm based on local rather than global linear relationship between samples. Finally, as expected, KSR-EN, as nonlinear extension of SR-EN, consistently outperforms all other imputation algorithms under both performance metrics. For example, the imputed results obtained by KSR-EN fit well with the true values according to Figure 3. For more clearly illustrating this, we present in Figure 4 the coefficient matrix W obtained by SR-EN and KSR-EN. Note that the sample index in the figure roughly reflects the proximity relation between samples. We can see that W derived from KSR-EN exhibits clear sparse and diagonal structure, indicating that in the high-dimensional feature space, each sample can be well represented as a linear combination of its neighboring samples within the same class. It also confirms that KSR-EN successfully discovers the underlying nonlinear structure of multiple subspaces. In contrast, the diagonal structure spreads in wider range in W obtained by SR-EN. For KSR-EN, we also notice from the figure of KSR-EN that some nonzero values in W appear far away from the main diagonal, seemingly violating our hypothesis. After careful analysis, we found that these off-diagonal nonzero values are mainly caused by the samples drawn from the intersection area of two arcs. Since the samples in this area are heavily mixed, it may be impossible to distinguish them completely.

### 4.3. Traffic Sensor Data Imputation

#### 4.3.1. Data Collection

**Figure 5:**
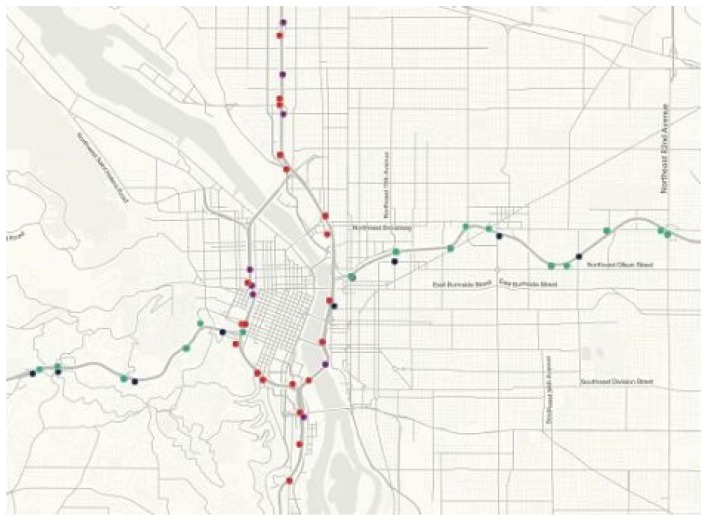
The selected sub-area road network of Portland, OR, USA.

**Figure 6:**
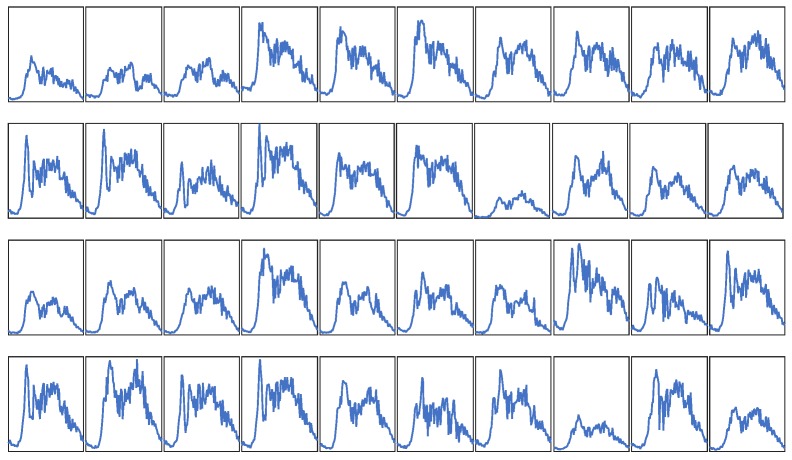
Illustration of traffic flow profiles from 40 sensors in the same day.

In this experiment, we evaluate the proposed KSR-EN algorithm on a real-world traffic flow dataset which is publicly accessible at http://portal.its.pdx.edu/. The data is collected from 40 loop detectors installed at Interstate 205 (I205) interstate highways. The selected sub-area road network is shown in Figure 5. The traffic volume is aggregated every 15 min and the unit is vehicles per 15 min (veh/15 min). For each loop detector, 96 data points is recorded per day. We use traffic data of 30 weekdays in the year of 2015 since the traffic flow profile on weekends and holidays are very different from weekdays. The total number of traffic volumes is 96 × 40 × 30 = 115,200. The whole data is organized as a 96 × 1200 matrix with each column denoting a sample. Figure 6 illustrates the traffic flow profiles captured by 40 sensors in the same day. As can be seen, despite of overall similarity, the variation of traffic flow at different road segments shows remarkable difference in terms of maximum traffic flow, the duration of rush hours, etc.

In order to comprehensively evaluate the recovery performance of different imputation algorithms under complex environments, we artificially inject MVs into original data by simulating three different missing patterns [2]. (i) Missing completely at random (MCAR), which means the presence or absence of MVs is completely independent of observed values and other parameters of interest. (ii) Missing at random (MAR), indicating that the occurrence of MVs depends on its neighboring points. (iii) Mixture of MCAR and MAR (MIXED), where half of MVs obey MCAR and the other half are from MAR. Generally speaking, MCAR is easier than MAR in the sense of recovery because MVs in the former case appear as isolated points randomly distributed, while MVs in the latter case often look like continuous missing, thus making accurate imputation more challenge. In addition, we also change the missing ratio δ, that is, the ratio of the number of missing entries to the total number of entries in data matrix, from 0.1 to 0.5 with step 0.1. Obviously, the larger δ, the harder the imputation task.

#### 4.3.2. Imputation Error

Table 2, Table 3 and Table 4 summarize the imputation errors obtained by different methods under MCAR, MAR, and MIXED missing patterns, respectively. The best results are highlighted in bold for clarity. From these results, we can obtain some interesting observations. As a typical local structure (instead of global structure) based approach, LLS is able to produce small imputation errors when the missing ratio is low. However, the performance deteriorates rapidly with increased missing ratio. It may be because that the reliable estimation of local structure or neighboring samples turns out to be very difficult given a set of samples with many unknown entries. Different from LLS, LRMC and PPCA all depend on the global linear subspace structure of data. PPCA works slightly better than LRMC on this dataset, and both outperform LLS when δ is larger than 0.3. However, these methods may fail to deliver good performance on the dataset with complex intrinsic structure. SR-EN, our proposed linear imputation approach, works better than the above conventional approaches, since it successfully accounts for the potential multiple linear subspace structure, thus avoiding the assumption of single subspace. KSR-EN further relax the restriction on linear subspace structures of SR-EN via effective nonlinear mapping from original input space to a high-dimensional feature space. By doing so, KSR-EN is able to explore multiple linear subspace structure in feature space, which is nonlinear in input space, and thus enhance the performance of SR-EN. As a result, KSR-EN is superior to SR-EN and the other competing approaches w.r.t. imputation errors, regardless of specific missing pattern or missing ratio. It suggests that the integration of nonlinear kernel mapping, as well as SR-EN, allows significant performance gain for the recovery of MVs in traffic scenarios.

#### 4.3.3. Influence of Parameters

**Figure 7:**
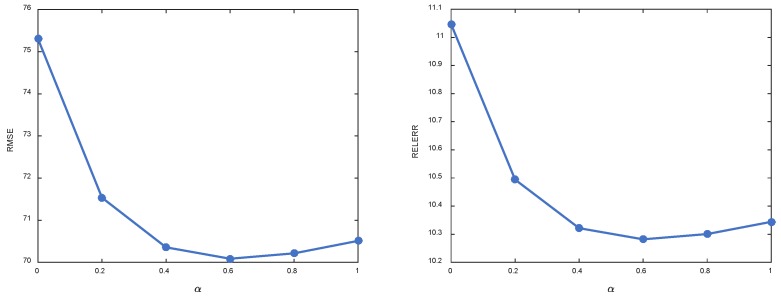
Performance variation of KSR-EN w.r.t. different values of α.

**Figure 8:**
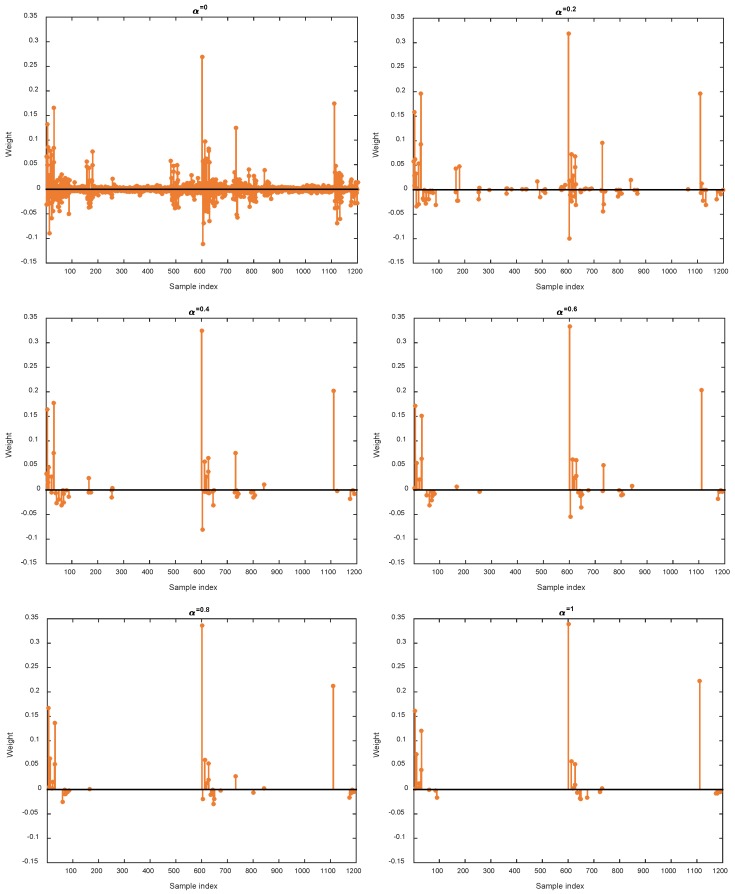
Variation of sparsity w.r.t. different values of α.

In this work, the linear combination of *l*_1_-norm and *l*_2_-norm, namely elastic net, is used as regularization to encourage highly correlated samples can be selected for reconstructing each target sample. As a result, the trade-off parameter α plays an indispensable role in the final model. On one hand, small α means that *l*_2_-norm dominates the regularization and makes the solution dense. On the other hand, large α will increase the portion of *l*_1_-norm and thus leads to solution with more sparsity. To this end, we first study the impact of parameter α. In this example, we focus on MCAR missing pattern and missing ratio *δ* = 0.2. To investigate the influence of α, we fix $C=2^{-5}$ and change α from 0 to 1 with step 0.2. The variation of RMSE and RELERR w.r.t. α is shown in Figure 7. Moreover, one sample is selected to show the variation of representation coefficients w.r.t. α. The results are shown in Figure 8. As we can see from Figure 7, small α and large α both degrade the imputation performance. From the viewpoint of sparsity, when α equals to zero, the resulting coefficients are dense. Large α tends to shrink many elements in the coefficients towards zero. We observe from extensive experiments that in most cases, optimal performance is achieved when α is between 0 and 1, thus confirming the effectiveness of elastic net regularization.

**Figure 9:**
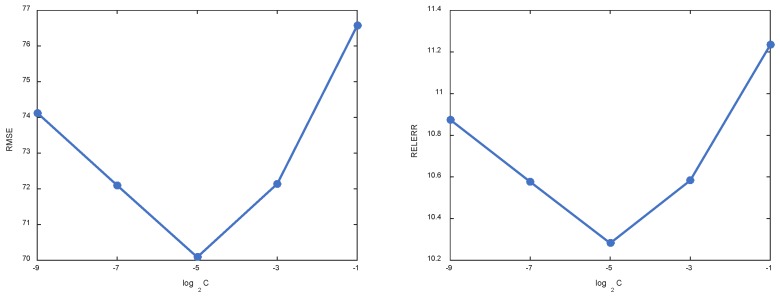
Performance variation of KSR-EN w.r.t. different values of C.

**Figure 10:**
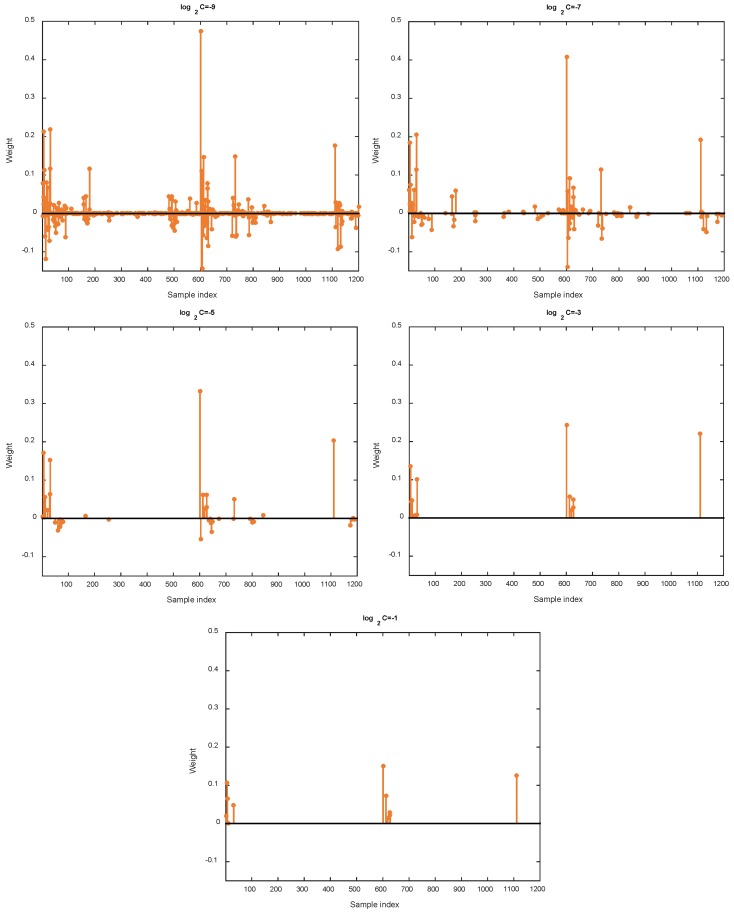
Variation of sparsity w.r.t. different values of C.

In what follows, we investigate the influence of trade-off parameter C. Through tuning parameter C, we can control the strength of regularization on coefficient matrix. Small C will weaken the role of regularization and leads to coefficient matrix with large magnitude values. It will make the resulting model fit well on the observed values but perform worse on other MVs. This is also known as overfitting in machine learning [52]. In contrast, large C tends to drive many coefficients to be small (or exact zero) and produces a model incapable of characterizing the inherent structure of data sufficiently. Similar to the above experiment, we fix α=0.6 and tune the value of C from $\left\{2^{-9},2^{-7},2^{-5},2^{-3},2^{-1}\right\}$. The variation of RMSE and RELERR is shown in Figure 9. Accordingly, the variation of representation coefficients of one selected sample is illustrated in Figure 10. We notice that when C is not too large or small, the obtained performance is satisfactory. This observation is consistent with the above analysis we made.

**Figure 11:**
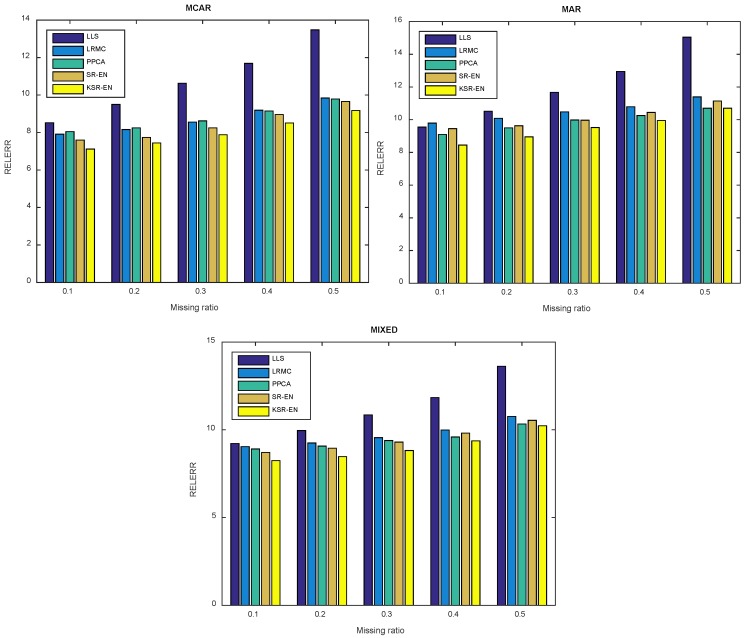
Relative error of each method with 30 min aggregation time.

Next, we investigate the influence of aggregation time on the imputation performance of each method. Larger aggregation time leads to smoother traffic flow profile, thus causing loss of detail information. Due to different aggregation time, the variation range of traffic flow in unit interval is much different. Therefore, relative error is more suitable when comparing imputation performance under different aggregation time. The aggregation time is set to be 30 min and the experimental results are shown in Figure 11. As can be seen, the proposed KSR-EN outperforms all the other approaches regardless of specific missing pattern or missing ratio, thus verifying the effectiveness of KSR-EN again.

#### 4.3.4. Computational Time

In this section, we report the computational time of different approaches employed in this paper. We take MIXED missing pattern as it is a combination of MCAR and MAR cases. The experimental results are shown in Table 5 as follows. As we can see, the proposed KSR-EN performs slower than other traditional approaches. It is well-known that sparse representation is a time-consuming procedure especially when the number of samples is large. In addition, gradient descent generally leads to slow convergence. To deal with this problem and improve the efficiency of our approach, several strategies can be exploited. For example, we can approximately solve sparse representation problem through greedy algorithms, such as matching pursuit (MP) or orthogonal MP (OMP) [53], etc. Alternatively, quasi-Newton approach can be used to replace the gradient descent, owing to its affordable memory request but fast convergence. We will concentrate on improving the efficiency of this model in the future work.

## 5. Conclusions

The problem of missing traffic sensor data imputation is studied in this work. Conventional sparse representation based imputation may lead to the loss of information conveyed in highly correlated samples. The application to real-world data with complex nonlinear structure is also problematic due to the drawbacks of the linear model. Therefore, we propose the KSR-EN model, which integrates elastic net regularization and the kernel method in a unified framework. In such a way, MVs imputation is performed in high-dimensional feature space rather than original input data space, benefiting accurate imputation. To solve the resulting model, an iterative algorithm is further developed by optimizing the representation coefficients and MVs alternatively. Experiments on both synthetic and traffic sensor data verify that exploiting nonlinear sparse representation, along with the combination of *l*_1_-norm and *l*_2_-norm, can provide more accurate imputation than other competing approaches.

In current work, MVs are estimated from a statistical perspective. Despite reduced estimation errors, the dynamic property of traffic flow is ignored in this work. Many traffic state estimation models have been developed using different techniques, such as extended Kalman filter (EKF) [13], Hamilton-Jacobi equations [12], etc. In future work, we will try to incorporate the traffic state estimation models into our MVs imputation approach to exploit the inherent relationship between variables. Another aspect deserving investigation is the theoretical supporting argument of our proposed model, such as the condition that accurate imputation can be guaranteed. We will focus on the problems in future work.

## Figures and Tables

**Table 1:** Imputation error root mean square error (RMSE) and relative error (RELERR) obtained by different approaches. The best results are highlighted in bold.

Metrics	Compared Methods			Ours	
	LLS	LRMC	PPCA	SR-EN	KSR-EN
RMSE	13.89 ± 1.30	56.05 ± 1.86	37.77 ± 1.55	25.07 ± 2.80	**7.00 ± 0.37**
RELERR	19.23 ± 1.85	77.60 ± 2.79	52.23 ± 2.24	34.68 ± 3.60	**9.69 ± 0.51**

**Table 2:** Imputation error RMSE and RELERR obtained by different approaches under missing completely at random (MCAR) missing pattern. The best results are highlighted in bold.

*δ*	Metrics	Compared Methods			Ours	
		LLS	LRMC	PPCA	SR-EN	KSR-EN
0.1	RMSE	76.62 ± 0.60	80.63 ± 0.86	79.06 ± 1.36	74.12 ± 1.22	**67.52 ± 0.43**
	RELERR	11.22 ± 0.09	11.80 ± 0.08	11.58 ± 0.15	10.85 ± 0.21	**9.89 ± 0.04**
0.2	RMSE	82.26 ± 0.70	83.30 ± 0.24	82.98 ± 0.24	76.43 ± 0.23	**70.65 ± 0.33**
	RELERR	12.06 ± 0.12	12.21 ± 0.05	12.16 ± 0.05	11.20 ± 0.02	**10.36 ± 0.06**
0.3	RMSE	89.94 ± 0.70	86.58 ± 0.74	85.47 ± 0.72	82.62 ± 1.84	**75.05 ± 0.96**
	RELERR	13.18 ± 0.08	12.69 ± 0.06	12.53 ± 0.06	12.11 ± 0.23	**11.00 ± 0.10**
0.4	RMSE	99.01 ± 0.56	90.94 ± 0.50	89.69 ± 0.63	85.83 ± 1.46	**80.42 ± 0.63**
	RELERR	14.49 ± 0.08	13.31 ± 0.04	13.13 ± 0.06	12.56 ± 0.19	**11.77 ± 0.07**
0.5	RMSE	113.03 ± 0.36	95.91 ± 0.44	95.83 ± 0.69	91.85 ± 1.46	**87.11 ± 0.43**
	RELERR	16.54 ± 0.04	14.04 ± 0.04	14.02 ± 0.07	13.46 ± 0.25	**12.75 ± 0.04**

**Table 3:** Imputation error RMSE and RELERR obtained by different approaches under missing at random (MAR) missing pattern. The best results are highlighted in bold.

*δ*	Metrics	Compared Methods			Ours	
		LLS	LRMC	PPCA	SR-EN	KSR-EN
0.1	RMSE	84.31 ± 1.11	98.31 ± 0.46	90.14 ± 0.88	87.44 ± 0.75	**77.84 ± 0.99**
	RELERR	12.00 ± 0.16	13.99 ± 0.10	12.83 ± 0.03	12.45 ± 0.22	**11.08 ± 0.20**
0.2	RMSE	91.89 ± 0.56	100.86 ± 1.17	95.13 ± 1.27	90.57 ± 1.41	**82.41 ± 0.97**
	RELERR	13.12 ± 0.04	14.40 ± 0.13	13.58 ± 0.17	12.93 ± 0.16	**11.76 ± 0.11**
0.3	RMSE	100.07 ± 1.51	103.06 ± 1.32	97.45 ± 1.28	94.56 ± 1.16	**87.63 ± 0.85**
	RELERR	14.34 ± 0.19	14.77 ± 0.15	13.96 ± 0.15	13.55 ± 0.16	**12.56 ± 0.12**
0.4	RMSE	112.54 ± 1.17	106.19 ± 0.79	101.50 ± 1.21	99.54 ± 0.73	**92.12 ± 1.73**
	RELERR	16.18 ± 0.17	15.26 ± 0.12	14.59 ± 0.16	14.31 ± 0.11	**13.24 ± 0.27**
0.5	RMSE	130.85 ± 1.96	109.78 ± 0.69	105.50 ± 0.87	104.55 ± 1.00	**99.21 ± 0.98**
	RELERR	18.84 ± 0.27	15.81 ± 0.11	15.19 ± 0.10	15.06 ± 0.15	**14.29 ± 0.14**

**Table 4:** Imputation error RMSE and RELERR obtained by different approaches under MIXED missing pattern. The best results are highlighted in bold.

*δ*	Metrics	Compared Methods			Ours	
		LLS	LRMC	PPCA	SR-EN	KSR-EN
0.1	RMSE	79.48 ± 1.67	89.57 ± 1.01	84.40 ± 2.17	80.48 ± 1.09	**71.98 ± 1.79**
	RELERR	11.46 ± 0.28	12.91 ± 0.18	12.17 ± 0.32	11.60 ± 0.18	**10.38 ± 0.29**
0.2	RMSE	86.60 ± 0.47	92.73 ± 0.05	89.37 ± 0.71	84.96 ± 1.44	**77.36 ± 1.38**
	RELERR	12.52 ± 0.12	13.40 ± 0.09	12.92 ± 0.07	12.28 ± 0.26	**11.18 ± 0.16**
0.3	RMSE	93.75 ± 0.86	95.18 ± 1.10	91.37 ± 1.22	87.88 ± 0.92	**81.06 ± 2.35**
	RELERR	13.58 ± 0.11	13.78 ± 0.13	13.23 ± 0.15	12.73 ± 0.14	**11.74 ± 0.31**
0.4	RMSE	104.31 ± 0.52	99.00 ± 0.57	95.26 ± 0.62	93.85 ± 0.71	**88.28 ± 1.68**
	RELERR	15.13 ± 0.06	14.36 ± 0.05	13.82 ± 0.09	13.61 ± 0.07	**12.80 ± 0.24**
0.5	RMSE	118.92 ± 1.31	103.14 ± 0.96	100.83 ± 0.65	98.33 ± 0.78	**94.02 ± 1.00**
	RELERR	17.28 ± 0.17	14.99 ± 0.12	14.65 ± 0.08	14.23 ± 0.10	**13.66 ± 0.12**

**Table 5:** Computational time(s) of different approaches.

*δ*	Compared Methods			Ours	
	LLS	LRMC	PPCA	SR-EN	KSR-EN
0.1	2.281	3.008	2.815	75.153	178.690
0.2	3.576	4.098	3.520	76.455	192.777
0.3	5.662	5.993	5.325	78.657	223.926
0.4	8.453	8.654	8.138	80.897	249.088
0.5	12.483	12.381	11.961	81.419	274.759
